# Mast Cell β-Tryptase Is Enzymatically Stabilized by DNA

**DOI:** 10.3390/ijms21145065

**Published:** 2020-07-17

**Authors:** Sultan Alanazi, Mirjana Grujic, Maria Lampinen, Ola Rollman, Christian P. Sommerhoff, Gunnar Pejler, Fabio Rabelo Melo

**Affiliations:** 1 Department of Medical Biochemistry and Microbiology, Uppsala University, 751 23 Uppsala, Sweden; Sultan.alanazi@imbim.uu.se (S.A.); mirjana.grujic@imbim.uu.se (M.G.); maria.lampinen@medsci.uu.se (M.L.); 2 Department of Medical Sciences, Uppsala University, 751 85 Uppsala, Sweden; ola.rollman@medsci.uu.se; 3 Institute of Laboratory Medicine, University Hospital, 80539 LMU Munich, Germany; sommerhoff@med.uni-muenchen.de; 4 Department of Anatomy, Physiology and Biochemistry, Swedish University of Agricultural Sciences, 756 51 Uppsala, Sweden

**Keywords:** tryptase, mast cells, DNA, heparin

## Abstract

Tryptase is a tetrameric serine protease located within the secretory granules of mast cells. In the secretory granules, tryptase is stored in complex with negatively charged heparin proteoglycans and it is known that heparin is essential for stabilizing the enzymatic activity of tryptase. However, recent findings suggest that enzymatically active tryptase also can be found in the nucleus of murine mast cells, but it is not known how the enzmatic activity of tryptase is maintained in the nuclear milieu. Here we hypothesized that tryptase, as well as being stabilized by heparin, can be stabilized by DNA, the rationale being that the anionic charge of DNA could potentially substitute for that of heparin to execute this function. Indeed, we showed that double-stranded DNA preserved the enzymatic activity of human β-tryptase with a similar efficiency as heparin. In contrast, single-stranded DNA did not have this capacity. We also demonstrated that DNA fragments down to 400 base pairs have tryptase-stabilizing effects equal to that of intact DNA. Further, we showed that DNA-stabilized tryptase was more efficient in degrading nuclear core histones than heparin-stabilized enzyme. Finally, we demonstrated that tryptase, similar to its nuclear localization in murine mast cells, is found within the nucleus of primary human skin mast cells. Altogether, these finding reveal a hitherto unknown mechanism for the stabilization of mast cell tryptase, and these findings can have an important impact on our understanding of how tryptase regulates nuclear events.

## 1. Introduction

Mast cells are immune cells originating from the bone marrow [1]. When they mature in local tissue environments, they acquire an abundance of secretory granules. These are filled with a plethora of preformed inflammatory mediators, including histamine, serotonin, cytokines, growth factors, lysosomal hydrolases, serglycin proteoglycan as well as large amounts of mast cell-restricted proteases [2]. The latter include tryptase, chymase and carboxypeptidase A3 [3,4].

Out of the mast cell proteases, β-tryptase (denoted tryptase in the following) is unique with respect to its tetrameric organization, where all of its active sites are facing a narrow, central pore [5]. Due to this organization, tryptase is resistant to all known physiological protease inhibitors, and has a relatively restricted substrate cleavage profile [5,6,7]. Previous work has shown that the storage of tryptase within the granules is critically dependent on serglycin, i.e., proteoglycans having strongly negatively charged glycosaminoglycan side chains of heparin (or chondroitin sulfate) type attached to the serglycin core protein [8,9]. In addition to promoting the storage of tryptase, it is known that heparin also is necessary for the assembly of enzymatically active tryptase tetramers [10,11]. Moreover, it has been shown that heparin is necessary for stabilizing the tetrameric, active organization of tryptase, and that the absence of stabilizing heparin is accompanied by a rapid loss of enzymatic activity [12].

In recent studies we have challenged the established notion of tryptase being confined to secretory granules, by showing that tryptase also can be found within the nucleus of murine mast cells, and also in human tumor cells exposed to human tryptase [13,14,15,16]. In the nucleus, tryptase was shown to proteolytically degrade several nuclear compounds such as histones and lamin B1, and we also showed that tryptase can regulate gene expression and proliferation [13,14,16]. Hence, these findings reveal that tryptase can be enzymatically active within the nuclear milieu and can have a functional impact on nuclear events. However, heparin is most likely not located within the nucleus and it is thereby not known how tryptase can maintain enzymatic activity in the nuclear compartment. Here we hypothesized that DNA could potentially substitute for heparin in stabilizing the active tryptase in the nuclear environment, the rationale being that DNA, similar to heparin, has a high negative charge density. Indeed, we showed that DNA has this capacity, hence providing a mechanism by which tryptase can execute enzymatic functions within the nucleus. 

## 2. Results

### 2.1. DNA Preserves the Enzymatic Activity of Tryptase 

**Figure 1:**
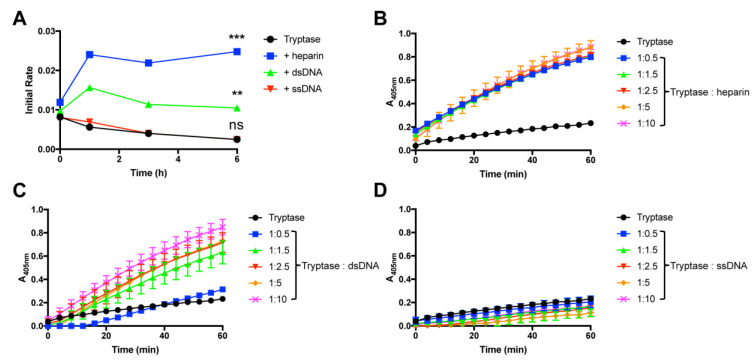
DNA stabilizes tryptase activity at room temperature. (**A**) β-tryptase (0.7 ng/µL in PBS, pH 7.2) was incubated at room temperature either alone or in the presence of either heparin, dsDNA or ssDNA (ratio 1:5) at the time periods indicated. Residual tryptase activity was then measured using the chromogenic substrate S-2288. (**B**–**D**) Dose response experiments for β-tryptase (0.7 ng/µL; in PBS, pH 7.2) after incubation for 3 h at room temperature, in the absence or presence of either heparin (**B**), dsDNA (**C**) or ssDNA (**D**) at the indicated concentrations. Residual tryptase activity was then measured using the chromogenic substrate S-2288. The data are representative of at least 3 individual experiments. Data are given as mean values ± SD (*n* = 3) ***, *p* ≤ 0.001 **, *p* ≤ 0.01; ns, not significant (One-way ANOVA).

To study mechanisms of tryptase stabilization we used human recombinant β-tryptase, produced in a yeast system [6]. When tryptase alone was incubated at room temperature in a neutral pH-buffer, we noted that it lost enzymatic activity in a time-dependent manner, with essentially a complete loss of activity seen after 6 h (Figure 1A). However, when heparin was included in the incubation mixture, the enzymatic activity was completely preserved during the time interval studied; in fact, the addition of heparin potentiated the tryptase activity in comparison with the baseline level (Figure 1A). To examine the possible tryptase-stabilizing effects of DNA, we used double stranded DNA (dsDNA) extracted from HMC-1 cells. As seen in Figure 1A, the addition of dsDNA also resulted in tryptase stabilization, with essentially complete preservation of enzymatic activity up to 6 h of incubation. Dose response experiments showed that, on a weight basis, heparin was more efficient than dsDNA in providing tryptase stabilization. However, with increasing concentration, dsDNA attained an equal tryptase-stabilizing effect as that of heparin (Figure 1B,C).

**Figure 2:**
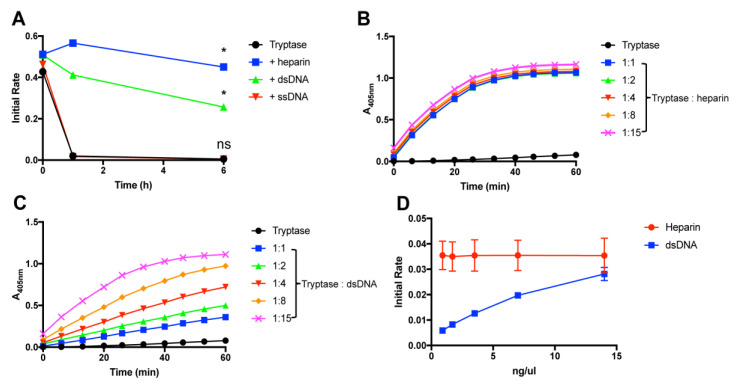
DNA stabilizes tryptase activity at 37 °C. (**A**) β-tryptase (1 ng/µL; in PBS, pH 7.2) was incubated at 37 °C either alone or in the presence of either heparin, dsDNA or ssDNA (ratio 1:5) at the time periods indicated. Residual tryptase activity was then measured using the chromogenic substrate S-2288. (**B, C**) Dose response experiments were performed where tryptase (1 ng/µL) was incubated for 3 h at 37 °C, in the absence or presence of either heparin, dsDNA or ssDNA at the indicated concentrations. (**D**) Initial reaction velocities from B–C are displayed. Residual tryptase activity was measured using the chromogenic substrate S-2288. The data are representative of at least 3 individual experiments. Data are given as mean values ± SD (*n* = 3) *, *p* ≤ 0.01; ns, not significant (One-way ANOVA).

In previous studies it has been shown that tryptase is considerably more labile in terms of enzymatic activity at physiological temperature than at room temperature [17]. Next, we therefore performed experiments to assess whether DNA has the ability to stabilize tryptase also at 37 °C. Indeed, these experiments showed that dsDNA conferred protection of tryptase activity also at physiological temperature, with almost equal efficiency as seen for heparin (Figure 2A). Dose response experiments showed that, on a weight basis, heparin was more efficient than dsDNA in providing tryptase stabilization (Figure 2B–D). However, with increasing concentration, DNA attained an equal tryptase-stabilizing effect as that of heparin (Figure 2D).

### 2.2. Single-Stranded DNA Is Not Capable of Preserving the Enzymatic Activity of Tryptase 

In dsDNA, the base pairing brings the negatively charged phosphoryl groups of the DNA backbone into close proximity, thereby resulting in a high charge density per area unit. This is in contrast to single-stranded DNA (ssDNA), where such approximation of negative charges is not prevalent. To assess whether the high charge density imposed by base pairing is important for tryptase stabilization, we next investigated the ability of ssDNA to stabilize tryptase. As seen in Figure 1A,D and Figure 2A, ssDNA (cDNA) did not show any detectable tryptase-stabilizing effects, suggesting that the high negative charge density of dsDNA is necessary for tryptase stabilization.

### 2.3. Fragmented dsDNA Possesses Tryptase-Stabilizing Activity

**Figure 3:**
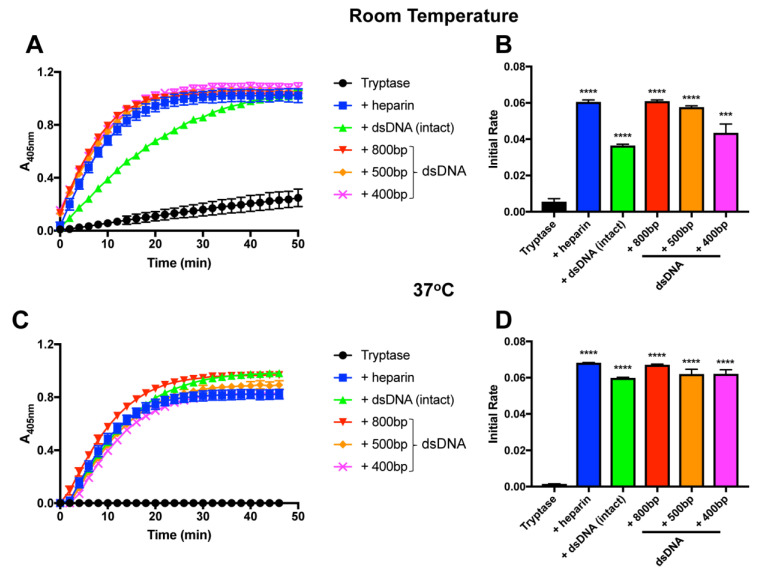
Fragmented DNA stabilizes tryptase activity. β-tryptase (0.07 µg) was incubated at room temperature (**A,B**) or 37 °C (**C,D**) in the absence or presence of either heparin, intact dsDNA or fragmented dsDNA of the indicated sizes. Residual tryptase activity was then measured using the chromogenic substrate S-2288. The data are representative of at least 3 individual experiments. Data are given as mean values ± SD (*n* = 3) *** *p* ≤ 0.001 **** *p* ≤ 0.0001 (Unpaired *t*-test).

In the next set of experiments, we assessed the size dependency of the stabilizing effect of dsDNA on tryptase. To this end, we fragmented dsDNA by sonication, and assessed the tryptase-stabilizing effects of dsDNA of different sizes. This showed that dsDNA fragments of sizes down to, at least, 400 base pairs had an equal stabilizing effect as that of intact dsDNA (Figure 3A–D).

### 2.4. Double-Stranded DNA Does Not Rescue the Enzymatic Activity of Inactivated Tryptase

**Figure 4:**
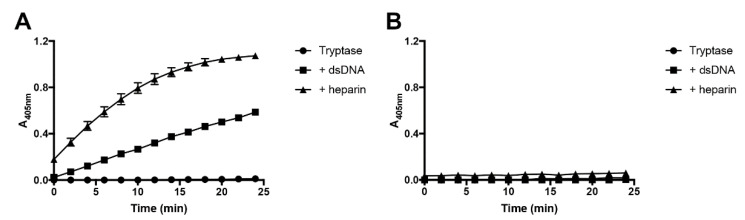
DNA or heparin do not rescue the activity of inactivated tryptase. (**A**) β-tryptase (0.07 μg) was incubated at 37 °C for 3 h in the absence or presence of either heparin or dsDNA, followed by measurement of residual tryptase activity using S-2288. (**B**) Human β-tryptase (0.07 μg) was enzymatically inactivated by incubation in the absence of heparin or dsDNA for 3 h (37 °C). After complete inactivation (3 h), tryptase was further incubated for 30 min, either alone or in the presence of either heparin or dsDNA as indicated. Tryptase activity was then measured using the chromogenic substrate S-2288. The data are representative of three independent experiments. Data are given as mean values ± SD (*n* = 3).

Next, we investigated whether heparin or dsDNA could rescue the loss of activity of inactivated tryptase. To this end, tryptase was first incubated for a time period sufficient to cause complete loss of enzymatic activity (Figure 4A). After inactivation, heparin or dsDNA were added to the tryptase, followed by the measurement of tryptase activity. As seen in Figure 4B, neither heparin nor DNA had the capacity to reverse the loss of activity in the absence of either of the stabilizing factors. 

### 2.5. DNA Promotes Histone Degradation by Tryptase

**Figure 5:**
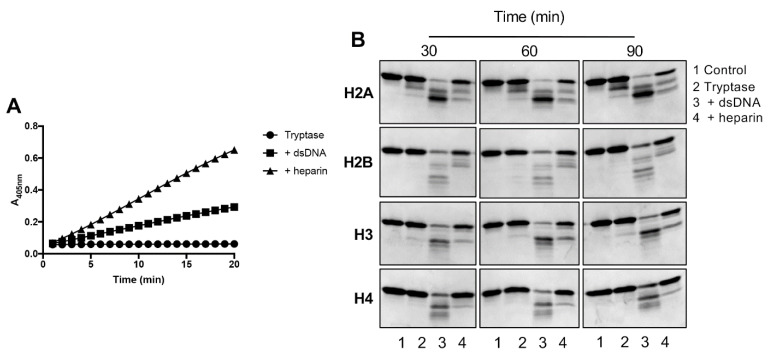
DNA-stabilized tryptase degrades core histones. β-tryptase (1 ng/µL) in PBS was incubated for 1 h at 37 °C, either alone or in the presence of either dsDNA (5 ng/µL) or heparin (5 ng/µL) as indicated; monitoring of residual enzymatic activity against the chromogenic substrate S-2288 is shown in (**A**). (**B**) Next, core histones (H2A, H2B, H3, H4; 260 ng) were added, followed by incubation for 30, 60 or 90 min (37 °C). Samples were then separated by SDS-PAGE, followed by visualization of bands by InstantBlue. The data are representative of three independent experiments.

In previous studies we showed that tryptase, located in the nucleus, has the capacity to cause histone degradation [13,14,16]. In the next set of experiments, we evaluated whether such tryptase-mediated histone degradation is affected by the presence of dsDNA. To verify the stabilizing effect of dsDNA and heparin on tryptase activity, we first measured their capacity to cleave the chromogenic substrate S-2288. In agreement with the results above, heparin-stabilized tryptase was more efficient in cleaving S-2288 than was the DNA-stabilized enzyme (Figure 5A). Next, we incubated the different core histones (H2A, H2B, H3, H4) with either tryptase alone, dsDNA-stabilized tryptase or heparin-stabilized tryptase, followed by SDS-PAGE analysis. These experiments showed that tryptase alone had minimal capacity to degrade any of the core histones (Figure 5B). In contrast, in the presence of either heparin or DNA, core histone degradation was seen (Figure 5B). Intriguingly though, all of the core histones were more efficiently degraded by dsDNA-stabilized vs. heparin-stabilized tryptase. The latter findings thus indicated that dsDNA-stabilized tryptase, despite having lower activity against S-2288 than that of heparin-stabilized tryptase, had a higher capacity to degrade core histones. It is also notable that the degradation of core histones was a rapid event, being completed within 30 min of incubation (Figure 5B).

### 2.6. Tryptase Is Located in the Nucleus of Human Mast Cells

**Figure 6:**
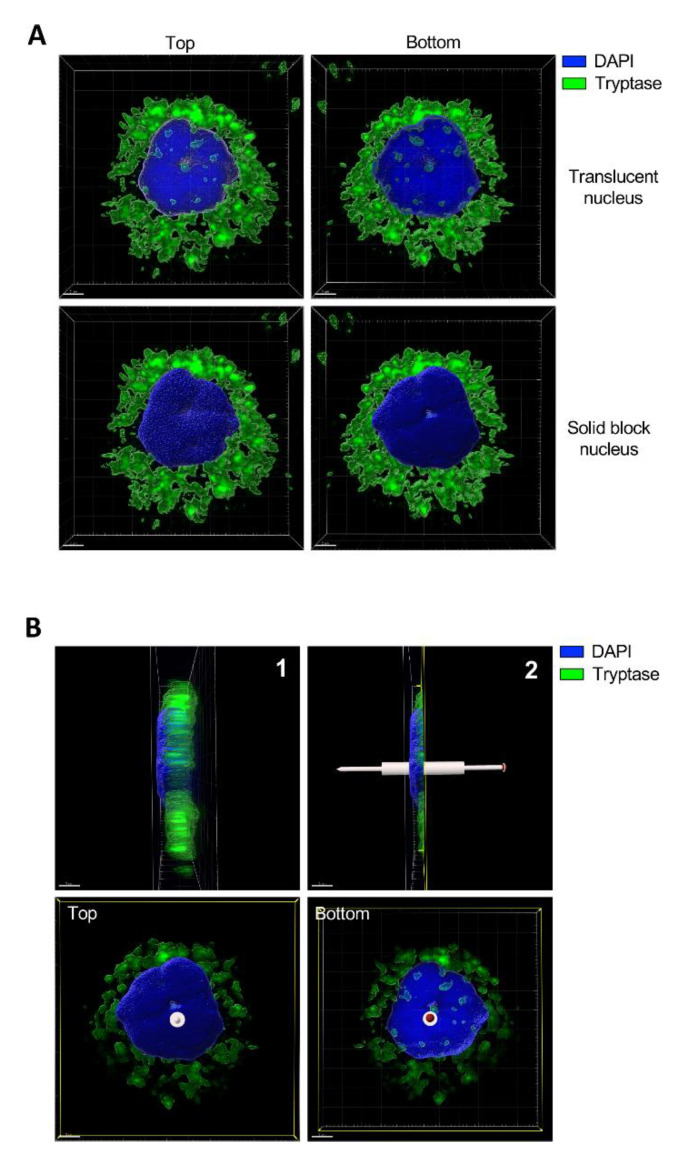
Tryptase is found in the nuclei of primary human skin mast cells. Human skin mast cells were stained for tryptase and with a nuclear dye (Hoechst33342), followed by confocal microscopy analysis. (**A**) 3-D view generated from Z-stack sections of a representative mast cell. Upper panels are showing top and bottom views from the same cell with a translucent nuclear structure. Note the presence of tryptase staining within the nucleus and that the staining is concealed when switching to solid block (lower panels), revealing that the tryptase is present within the nuclear compartment. Abundant tryptase staining is seen in the cytoplasm, representing secretory granules. (**B**) 1: Right side view from A (lower right panel). 2: Cross-section cut dividing the nucleus in half. Lower panels are showing top and bottom views from panel B2. Note that tryptase is present in different layers of the nucleus. The staining was performed twice and panels are showing a representative cell from more than ten individual cells analyzed.

In previous studies we have shown that tryptase can be found within the nucleus of mouse mast cells [13,14]. However, the presence of tryptase in the nucleus of human mast cells has not been investigated. To address this, we double-stained mast cells purified from human eyelid skin with a nuclear marker (Hoechst33342) and for tryptase, followed by confocal microscopy analysis. As seen in Figure 6A, tryptase staining was, as expected, abundant in granule-like compartments widely distributed within the cytosol of the mast cells. In addition, the confocal microscopy analysis revealed the presence of tryptase in the nucleus. To provide proof for the intranuclear presence of tryptase, images were generated with both translucid and solid block-shifted visualization of Hoechst33342 staining. With the solid block-shifted images, staining associated with the surface of the nucleus will be visible whereas staining within the nuclear core would be blocked. On the contrary, the translucid Hoechst33342 images will reveal positivity at all layers of the nucleus. As seen in Figure 6A, clear tryptase positivity was seen when adopting the translucid staining, whereas tryptase positivity was undetectable in the solid block-shifted images. This supports the notion that tryptase is located within the nuclear body, rather than being associated with the surface of the nucleus. Figure 6B shows a side view of the nucleus, revealing that tryptase is present in multiple layers of the nucleus.

## 3. Discussion

There is to date relatively little reported evidence for the presence of any proteolytic enzymes residing in the nucleus of mammalian cells. Essentially, this notion is limited to findings showing that cathepsin B and legumain can be found in the nucleus of cancer cells [18,19] and there is also data showing that cathepsin L can be localized in the nucleus of embryonic stem cells [20]. In addition, we showed previously that one of the mast cell-restricted proteases, tryptase, can be located in the nucleus of murine mast cells [13] and we also demonstrated that tryptase can be taken up to the nuclei of melanoma cells [16]. There is also very little insight into the functional impact of proteases located within the nuclei of cells. In studies of embryonic stem cells, evidence suggested that nuclear cathepsin L has the ability to truncate core histones (H3), and it was inferred that this could have epigenetic consequences by erasing histone marks deposited on the N-terminal ends of H3 [20]. As proof of this concept, we showed recently that nuclear tryptase cleaves off certain epigenetic marks, and we showed that this had a profound effect on the gene expression profile of mast cells [14]. In addition, we demonstrated that tryptase entering the nucleus of tumor cells has the ability to truncate H3 [16].

These studies have established that tryptase can be found in the nucleus in an enzymatically active form. However, a key question is to understand how tryptase can maintain enzymatic activity in the nuclear milieu. Generally, tryptase, being resistant to endogenous protease inhibitors [5,6], is well adapted to carry out functions in environments rich in protease inhibitors, such as the cytosol and plasma. However, tryptase is known to be dependent on heparin for activation and stabilization [10,11,12]. There is to date no evidence to suggest that heparin, or similar highly charged glycosaminoglycans are found in the nuclei of mast cells or tumor cells. It would thereby be assumed that tryptase, after entering the nucleus, rapidly loses enzymatic activity. However, since this is apparently not the case, we hypothesized that alternative tryptase-stabilizing mechanisms might prevail in the nuclear milieu. 

Here we assessed the possibility that tryptase can in fact be stabilized by interaction with dsDNA and, indeed, we present evidence in support of this notion. This study thereby provides a plausible scenario explaining how tryptase located in the nucleus of various cell types can be enzymatically active. When comparing the stabilizing potential of dsDNA with the canonical tryptase ligand, i.e., heparin, we showed that heparin is somewhat more efficient than dsDNA in promoting tryptase stabilization (with regard to activity towards a chromogenic substrate), on a weight basis. However, when increasing the dsDNA:tryptase ratio we found that tryptase attained equal maximal activity as when stabilized with heparin. Since the concentration of dsDNA in the nucleus is exceptionally high, it is thus likely that this milieu confers optimal stabilization conditions for tryptase activity. Further, we demonstrated that dsDNA-stabilized tryptase, similar to tryptase stabilized by heparin, efficiently degrades core histones. Hence, this finding is also in line with our previous findings showing that tryptase has the capacity to truncate various core histones [13,16], and it is thus plausible that the interaction of nuclear tryptase with dsDNA enables this impact of tryptase. Intriguingly though, dsDNA-and heparin-stabilized tryptase were observed to have differential impacts on the core histones, with dsDNA-stabilized tryptase being more effective in degrading the core histones than the heparin-stabilized enzyme. The biological impact of this difference is at present unclear. However, we may speculate that tryptase stabilized by dsDNA could have a higher ability than heparin-stabilized tryptase to erase epigenetic marks residing on the N-terminals of the respective core histones.

We also demonstrated that tryptase can be found in the nucleus of human mast cells. Hence, this adds to our previous findings where the focus has been on murine mast cells, and on the uptake of tryptase into the nucleus of tumor cells. Altogether, this supports the notion that nuclear tryptase can have a functional impact on mast cells, and that mechanisms serving to stabilize tryptase in the nuclear milieu can have an important function to regulate such nuclear activities of tryptase. However, we cannot at present explain the exact mechanism leading to the entry of tryptase into the nucleus.

From a different angle, we may also speculate that DNA could have an extracellular function as a tryptase stabilizer. For example, in situations when DNA is released to the exterior in the form of extracellular traps, either from mast cells or other immune cells such as neutrophils, tryptase could be entangled in such structures and may become enzymatically stabilized by interaction with the DNA. Tryptase could thus account for proteolytic activity in extracellular traps, which could have an impact on, e.g., bacterial clearance or tumor settings [21]. Another scenario could be that tryptase, released from activated mast cells during inflammatory conditions, can interact with DNA released from necrotic cells. Such an interaction could serve to stabilize extracellular tryptase, and may account for proteolytic activity that could regulate the levels of various pro-inflammatory compounds known to be substrates for tryptase, such as TSLP, IL-21, CCL7, CCL19 or eotaxin [22].

## 4. Materials and Methods

### 4.1. Reagents

Recombinant human β-tryptase was prepared as described [6]. Recombinant human histones (H2A, H2B, H3, H4) were purchased from New England Biolabs (Ipswich, MA, USA). Porcine intestinal mucosa heparin was from Sigma-Aldrich (Steinheim, Germany). Rabbit anti-histone H2A, H2B, H3 and H4 polyclonal antibodies were from Abcam (Cambridge, UK).

### 4.2. cDNA Preparation

Single-stranded cDNA was prepared using mRNA extracted from HMC-1 cells with an iScript cDNA Synthesis Kit, following the manufacturer’s instructions, and the cDNA concentration and purity were measured using a NanoDrop-1000 Spectrophotometer (Thermo Fisher Scientific, Wilmington, SC, USA).

### 4.3. Tryptase Activity

Tryptase (0.07 µg) in phosphate-buffered saline PBS (pH 7.2; 100 µL total volume) was incubated either alone or in the presence of variable concentrations of heparin, dsDNA (double-stranded), fragmented dsDNA or ssDNA. Incubations were performed in 96-well plates. The plates were incubated at either 37 °C (5% CO_2_) or at room temperature. At various time points, absorbance was read after adding 20 µL from a stock solution (10 mM in H_2_O) of the chromogenic substrate S-2288 (Chromogenix, Milano, Italy). Tryptase activity was monitored by reading the absorbance at 405 nm over 60 min using a microplate reader (M200-TECAN Infinite). Assays were performed in triplicate and were repeated at least twice.

### 4.4. DNA Analysis

DNA was isolated from HMC-1 cells using a QIAamp DNA Mini Kit, following the manufacturer’s instructions. For gel preparation, we used 1.5% agarose gels in TAE (Tris-acetate-EDTA) buffer (*w*/*v*), with 0.1 μL/mL of Gel Red Nucleic Acid dye (Biotium, Inc., Fermont, CA, USA). The samples were diluted in 1 × gel loading dye (0.25% bromphenol blue, 0.25% xylene cyanol, 30% glycerol). The loading for each sample was 10 µL (20 µg of DNA) and agarose gels were run at 100V. To prepare fragmented DNA, the DNA was diluted in PBS (10 ng/µL) and sonicated by using a Bioruptor^®^ Plus sonication device (Diagenode, Denville, NJ, USA) with cycle conditions adjusted to 30 s on/30 s off for 5, 10, and 15 cycles. 

### 4.5. Histone Degradation

Recombinant human tryptase (1 ng/µL) in PBS, was incubated at 37 °C for 1 h either alone or in the presence of either heparin or double-stranded DNA (5 ng/µL). Aliquots were taken and frozen quickly and tryptase activity was further monitored in a microplate reader (M200-TECAN infinite) by monitoring absorbance at 405 nm (using the S-2288 assay) for 20 min. 2 µL of recombinant human core histones 2A (H2A), H2B, H3 or H4 (130 ng/µL) were added individually to each reaction tube containing unused aliquots from the previous reaction, followed by incubation for 30 min. The reactions were stopped by adding Laemmli SDS-PAGE sample buffer followed by heating at 95 °C for 5 min. SDS-PAGE was performed using 4–20% mini-protean tgx stain-free gels (BioRad, Hercules, CA) and visualization of protein bands was performed with a Coomassie based staining solution for protein gels (InstantBlue, Expedeon, Cambridgeshire, UK). 

### 4.6. Purification and Culture of Human Skin Mast Cells

Human skin mast cells were isolated as described previously with some modifications [23]. Eyelid skin was obtained from cosmetic surgeries with informed consent of the patients. Eyelids from two individuals were combined and used for one experiment. The skin was cut into stripes and treated with dispase (Roche Diagnostics Scandinavia AB) at 0.5 mg/mL, 4 °C overnight. Thereafter, the epidermis was removed from the dermis, the latter chopped into small pieces and digested with collagenase type 2 at 1.5 mg/mL (Sigma-Aldrich, Saint Louis, MI, USA), hyaluronidase type-1S at 0.75 mg/mL (Sigma-Aldrich, Saint Louis, MI, USA) and DNAse at 10 µg/mL (VWR, Radnor, PE, USA) for 1.5 h at 37 °C under continuous shaking. Isolated cells were separated from the remaining tissue by three steps of filtration (pore sizes 250, 100, and 30 μm). Mast cells were further purified from cell suspensions by positive selection using anti-human c-Kit microbeads (Miltenyi Biotec, Bergisch Gladbach, Germany), and a magnetic cell sorter separation device. Mast cell purity in the preparations typically exceeded 95%, as assessed by toluidine blue. Skin mast cells were cultured in Basal Iscove’s medium (Gibco/Thermo Fisher Scientific, Waltham, MA, USA), supplemented with 10% heat-inactivated fetal calf serum (FCS), 4 mM l-glutamine, and antibiotics, and were processed for downstream applications, as described below.

### 4.7. Immunofluorescence and Laser-Scanning Microscopy

Aliquots of 200 μL from 0.3 × 10^6^ cells/mL of freshly isolated human skin mast cells suspensions were dropped into round areas on microscopic glasses made with a liquid-repellent slide marker pen. The suspension was kept for 15 min and liquid was removed carefully with filter paper. The remaining cells were left to dry for 15 min. Samples were fixed and permeabilized with methanol for 10 min, followed by a 15 min drying step. On the top of each area, 100 μL of the mouse monoclonal human mast cell tryptase antibody (Abcam, Cambridge, UK) (1:500) in tris-buffered saline TBS 1% bovine serum albumin BSA and/or isotype control at the same concentration were left in contact with cells overnight at 4 °C, followed by three times washing with TBS-T. A total of 100 μL of anti-mouse Alexa-488 conjugated antibody in TBS 1% BSA was added to each slide. The slides were kept at room temperature in the dark for 2 h and washed three times with TBS-T. Finally, Hoechst33342 (NucBlue, Life Technologies, Carlsbad, CA) was added, followed by three times washing with TBS-T. The slides were mounted with SlowFade^TM^ diamond antifade mounting medium (Invitrogen, Eugene, OR, USA) and cover glass. Samples were analyzed using a laser-scanning microscope equipped with ZEN 2009 software (LSM 710; Carl Zeiss, Berlin, Germany). 

### 4.8. Statistical Analysis

The statistical significance between groups was determined by using One-way ANOVA and unpaired *t*-test. All graphs were prepared, and statistics calculated, using GraphPad Prism 7.0 (GraphPad software Inc., San Diego, CA). A *p*-value of less than 0.05 was considered significant. 

## References

[1] Gurish M.F., Austen K.F. (2012). Developmental origin and functional specialization of mast cell subsets. Immunity.

[2] Wernersson S., Pejler G. (2014). Mast cell secretory granules: Armed for battle. Nat. Rev. Immunol..

[3] Pejler G., Abrink M., Ringvall M., Wernersson S. (2007). Mast cell proteases. Adv. Immunol..

[4] Pejler G., Ronnberg E., Waern I., Wernersson S. (2010). Mast cell proteases: Multifaceted regulators of inflammatory disease. Blood.

[5] Pereira P.J., Bergner A., Macedo-Ribeiro S., Huber R., Matschiner G., Fritz H., Sommerhoff C.P., Bode W. (1998). Human beta-tryptase is a ring-like tetramer with active sites facing a central pore. Nature.

[6] Sommerhoff C.P., Bode W., Pereira P.J., Stubbs M.T., Sturzebecher J., Piechottka G.P., Matschiner G., Bergner A. (1999). The structure of the human betaII-tryptase tetramer: Fo(u)r better or worse. Proc. Natl. Acad. Sci. USA.

[7] Hallgren J., Pejler G. (2006). Biology of mast cell tryptase. An inflammatory mediator. FEBS J..

[8] Forsberg E., Pejler G., Ringvall M., Lunderius C., Tomasini-Johansson B., Kusche-Gullberg M., Eriksson I., Ledin J., Hellman L., Kjellen L. (1999). Abnormal mast cells in mice deficient in a heparin-synthesizing enzyme. Nature.

[9] Abrink M., Grujic M., Pejler G. (2004). Serglycin is essential for maturation of mast cell secretory granule. J. Biol. Chem..

[10] Hallgren J., Karlson U., Poorafshar M., Hellman L., Pejler G. (2000). Mechanism for activation of mouse mast cell tryptase: Dependence on heparin and acidic pH for formation of active tetramers of mouse mast cell protease 6. Biochemistry.

[11] Hallgren J., Spillmann D., Pejler G. (2001). Structural requirements and mechanism for heparin-induced activation of a recombinant mouse mast cell tryptase, mouse mast cell protease-6: Formation of active tryptase monomers in the presence of low molecular weight heparin. J. Biol. Chem..

[12] Schwartz L.B., Bradford T.R. (1986). Regulation of tryptase from human lung mast cells by heparin. Stabilization of the active tetramer. J. Biol. Chem..

[13] Melo F.R., Vita F., Berent-Maoz B., Levi-Schaffer F., Zabucchi G., Pejler G. (2014). Proteolytic histone modification by mast cell tryptase, a serglycin proteoglycan-dependent secretory granule protease. J. Biol. Chem..

[14] Melo F.R., Wallerman O., Paivandy A., Calounova G., Gustafson A.M., Sabari B.R., Zabucchi G., Allis C.D., Pejler G. (2017). Tryptase-catalyzed core histone truncation: A novel epigenetic regulatory mechanism in mast cells. J. Allergy Clin. Immunol..

[15] Grujic M., Hellman L., Gustafson A.M., Akula S., Melo F.R., Pejler G. (2020). Protective role of mouse mast cell tryptase Mcpt6 in melanoma. Pigment Cell Melanoma Res..

[16] Rabelo Melo F., Santosh Martin S., Sommerhoff C.P., Pejler G. (2019). Exosome-mediated uptake of mast cell tryptase into the nucleus of melanoma cells: A novel axis for regulating tumor cell proliferation and gene expression. Cell Death Dis..

[17] Hallgren J., Lindahl S., Pejler G. (2005). Structural requirements and mechanism for heparin-dependent activation and tetramerization of human betaI- and betaII-tryptase. J. Mol. Biol..

[18] Haugen M.H., Johansen H.T., Pettersen S.J., Solberg R., Brix K., Flatmark K., Maelandsmo G.M. (2013). Nuclear legumain activity in colorectal cancer. PLoS ONE.

[19] Tedelind S., Poliakova K., Valeta A., Hunegnaw R., Yemanaberhan E.L., Heldin N.E., Kurebayashi J., Weber E., Kopitar-Jerala N., Turk B. (2010). Nuclear cysteine cathepsin variants in thyroid carcinoma cells. Biol. Chem..

[20] Duncan E.M., Muratore-Schroeder T.L., Cook R.G., Garcia B.A., Shabanowitz J., Hunt D.F., Allis C.D. (2008). Cathepsin L proteolytically processes histone H3 during mouse embryonic stem cell differentiation. Cell.

[21] Cedervall J., Zhang Y., Huang H., Zhang L., Femel J., Dimberg A., Olsson A.K. (2015). Neutrophil Extracellular Traps Accumulate in Peripheral Blood Vessels and Compromise Organ Function in Tumor-Bearing Animals. Cancer Res..

[22] Fu Z., Akula S., Thorpe M., Hellman L. (2019). Highly Selective Cleavage of TH2-Promoting Cytokines by the Human and the Mouse Mast Cell Tryptases, Indicating a Potent Negative Feedback Loop on TH2 Immunity. Int. J. Mol. Sci..

[23] Babina M., Guhl S., Starke A., Kirchhof L., Zuberbier T., Henz B.M. (2004). Comparative cytokine profile of human skin mast cells from two compartments—Strong resemblance with monocytes at baseline but induction of IL-5 by IL-4 priming. J. Leukoc. Biol..

